# Toward Security Enhanced Provisioning in Industrial IoT Systems

**DOI:** 10.3390/s18124372

**Published:** 2018-12-10

**Authors:** Sungmoon Kwon, Jaehan Jeong, Taeshik Shon

**Affiliations:** 1 Department of Computer Engineering, Ajou University, 206, World cup-ro, Yeongtong-gu, Suwon-si, Gyeonggi-do 16499, Korea; calmcombat@gmail.com (S.K.); qnpfr7179@gmail.com (J.J.); 2 Department of Cybersecurity, Ajou University, 206, World cup-ro, Yeongtong-gu, Suwon-si, Gyeonggi-do 16499, Korea

**Keywords:** IIoT, ISA 100.11a, IWSN, provisioning, security

## Abstract

Through the active development of industrial internet of things (IIoT) technology, there has been a rapid increase in the number of different industrial wireless sensor networks (IWSNs). Accordingly, the security of IWSNs is also of importance, as many security problems related to IWSN protocols have been raised and various studies have been conducted to solve these problems. However, the provisioning process is the first step in introducing a new device into the IIoT network and a starting point for IIoT security. Therefore, leakage of security information in the provisioning process makes exposure of secret keys and all subsequent security measures meaningless. In addition, using the exploited secret keys, the attacker can send false command to the node or send false data to the network manager and it can cause serious damage to industrial infrastructure depending on the IWSN. Nevertheless, a security study on the provisioning process has not been actively carried out, resulting in a provisioning process without guaranteed security. Therefore, in this paper, we analyzed security issues of the provisioning process in IWSN by researching prominent IWSN standards, including ISA 100.11a, WirelessHART, and Zigbee, and also an ISA 100.11a-certified device and provisioning process-related studies. Then, we verified the security issues of the provisioning process through testing and analyzing the provisioning process using the ISA 100.11a standard-implemented devices and ISA 100.11a-certified devices. Finally, we discuss security considerations and the direction of future research on provisioning security for IWSN in the IIoT era.

## 1. Introduction

In recent industrial control systems, the Internet of Things (IoT) has been introduced to manage large areas efficiently or to manage dangerous or difficult to access areas. IoT that meets the requirements of robustness, reliability, latency, and jitter required in industrial control systems is called the Industrial Internet of Things (IIoT).

The Industrial Internet Consortium (IIC) is an organization in which various industrial control systems and IoT vendors participate in developing IIoT technology. Each year since its establishment in 2014, the IIC has published IIoT demonstration cases and related research. In particular, the IIoT demonstrations have had a great effect on industrial control systems by constructing test beds and conducting tests for various fields, such as energy, healthcare, manufacturing, transportation, and security, which has resulted in the introduction of IIoT systems in various industrial control system fields.

In the IIoT system, the wireless sensor network (WSN) used to connect sensor nodes to various environments is called the industrial wireless sensor network (IWSN). The widely used IWSN protocols are Zigbee, WirelessHART, and ISA 100.11a, and these protocols are based on IEEE 802.15.4 [1] (low-rate wireless personal area networks (LR-WPANs) standard). Zigbee is the first standard that implements the upper layer based on IEEE 802.15.4, and its latest standard is Zigbee PRO [2]. However, Zigbee does not support channel hopping, which is used for robustness against external interference in industrial control systems, and has a disadvantage in that it does not have enough scalability to support large topologies [3]. Nevertheless, various IWSN studies using Zigbee such as [4,5,6,7] have been conducted even up to recently. WirelessHART [8] is an extended standard for the wireless communication of equipment using the highway addressable remote transducer (HART) protocol, which has been used since the 1980s. WirelessHART is the first IWSN international standard approved by the international electrotechnical commission (IEC). While Zigbee and WirelessHART use existing standards or equipment in WSN, ISA 100.11a is an IWSN-specific standard designed for IWSN that considers the characteristics of both industrial control systems and wireless sensor networks. IEC 62734 [9] is the latest standard of ISA 100.11a, and was approved by the IEC in 2014 through the first publication of the standard in 2009. Since the publication of the ISA 100.11a standard, it has been used in various industrial control system fields, such as chemicals, life science, mining, oil and gas, refining, and pulp and paper [10].

In order to apply the IWSN standard to industrial control systems, security for the IWSN is essential. Therefore, many studies have been carried out to identify vulnerabilities through security analysis of the IWSN standard and to find countermeasures [11,12,13,14,15,16,17]. While it is true that the security of the IWSN standard has been strengthened through these studies, there is still a lack of study on the provisioning process, which is where all security begins. The provisioning process is a phase of obtaining network information and secret information necessary for a new device to join an existing IWSN. Beginning at the network join process, various keys are generated by using the secret information transmitted in the provisioning process. If the information transmitted during the provisioning process is leaked, the attacker can obtain various secret keys through eavesdropping, so the security of all the subsequent phases becomes meaningless. In addition, using exploited secret keys, the attacker can send false command from the network manager to the node or send false data from the node to the network manager. IWSN is used in various fields including chemicals, health care, life science, mining, oil and gas, refining and factory. Therefore if nodes have management of process functions, an attacker can directly cause sabotage to industrial infrastructure by sending false commands. Even if nodes don’t have management of process functions, there are scenarios such as when sensing nodes collect information for human safety, where an attacker can cause casualties by sending false sensing data. Nevertheless, an appropriate security mechanism for the provisioning process cannot be provided for environments with some security constraints, such as an environment in which an authentication system for a certificate is not built, and the matters of security are left to the user. Therefore, the provisioning process could be the main target of attackers because it is relatively more security-vulnerable than the subsequent phases which have higher cryptographic security mechanisms. If the attack is effective, any strong security for the subsequent phases will be meaningless. Therefore, the security of the provisioning phase should be treated as an important consideration in the IWSN configuration.

In this paper, we make the following contribution through conducting study to enhance the security of the provisioning process of the IWSN. We analyze the security issues in the provisioning methods of IWSN standards, including Zigbee, WirelessHART and ISA 100.11a, and also ISA 100.11a certified devices. To verify the security issues of the provisioning methods, an experimental environment for the provisioning methods is constructed and verification is carried out through experiments on the provisioning methods of the ISA 100.11a standard and the certified devices. Based on the results of the experimental analysis and comparison, we will build a knowledge base which may be usable for future research to build IWSNs and enhance security.

The organization of this paper is as follows: Section 2 describes the security studies on IWSN standards. Section 3 explains the security issues of the provisioning process by analyzing the provisioning process in the IWSN standards, ISA 100.11a-certified devices and related studies on provisioning security. We also suggest measures usable for responding to the security issues noted. In Section 4, the contents of Section 3 are verified through experiments and conclusions are made in Section 5.

## 2. Related Work

Various IWSN security studies have been carried out that have contributed to the security development of the IWSN standard. The studies include those that analyze IWSN security based on the IWSN standard, the IWSN standard’s vulnerability and countermeasures, security by applying WSN and other threat models to IWSN, and those that research applying IWSN security techniques. The following is a description of the research related to IWSN security.

Willing [11] and Nixon and Rock [12] analyze the security of the IWSN standard based on the IWSN standard documents, such as Zigbee, WirelessHART, and ISA 100.11a. Willing analyzes the Zigbee and ISA 100.11a standards and describes issues for applying IEEE 802.15.4-based Zigbee and ISA 100.11a to the industrial environment. Nixon et al. analyzed and compared the security functions of WirelessHART and ISA 100.11a standards and described the advantages and disadvantages of the security functions of each standard. Studies by Raza et al. [13] and Olawumi et al. [14] proposed possible threats and countermeasures by analyzing the vulnerabilities of the IWSN protocol. Raza et al. presented 13 possible threats and mitigations through the security analysis of the WirelessHART protocol. Olawumi et al. showed three types of effective attacks, using vulnerabilities of the Zigbee standard, and proposed countermeasures against them. Alcaraz and Lopez [15] and Bayou et al. [16] applied threat models of other networks to the IWSN. Alcaraz and Lopez analyzed the application of the supervisory control and data acquisition (SCADA) threat model and the WSN threat model in Zigbee, WirelessHART, and ISA 100.11a network environments and proposed countermeasures. Bayou et al. proposed an intrusion detection deployment scheme for IWSNs by applying existing WSN vulnerabilities to IWSNs. In order to apply the security of the IWSN, Jung et al. [17] conducted a study of effective key updates in ISA 100.11a. Various studies have been carried out to analyze IWSN standards, vulnerabilities of IWSNs, application of WSNs and other threat models, and applying IWSN security techniques, all of which have contributed to the development of IWSN standard security.

Studies have also been conducted on the security of the provisioning process, which is not only the first step for a new device to participate in IWSN but also the first step of IWSN security, such as those by Wang [18], Park and Lee [19], and Chen et al. [20]. Wang analyzed the provisioning process of ISA 100.11 and WirelessHART in detail, examined the security of the provisioning process, and explained cases having security issues. Park and Lee proposed the IEEE 802.15.4-based provisioning process, which uses elliptic curve Diffie–Hellman (ECDH)-based authentication and encryption techniques. Chen et al. applied the securing network access method for WSNs proposed by Sun et al. [21] to WirelessHART. Wang noted that the IWSN standard does not provide a suitably secure provisioning process for various IWSN environments and requires more research. There is a serious lack of the research for the provisioning process compared to the research for other IWSN security elements. In addition, the technique proposed by Park and Lee does not require a certificate authority (CA) for authentication, but has a restriction to share the ECDH public key in advance. The technique proposed by Chen requires CA-related information based on a public key certificate in advance, and provisioning to a suitable network is impossible when multiple IWSNs are used.

The pre-distribution problem, such as that of provisioning, is not new. For the WSN, there are already many studies that address the pre-distribution problem. Mian et al. [22] suggested an effective authentication scheme for WSNs, and Arcangelo et al. [23] analyzed some state-of-the-art key pre-distribution schemes and suggested an improved scheme. Nonetheless, the scheme of Mian et al. needs a pre-shared key, and other state-of-the-art schemes, including that of Arcangelo et al., also need some preloaded key material. Without information such as a pre-shared key and preloaded key material, these schemes cannot work and the provisioning process is a step for preloading this kind of information. Therefore, in this paper we will analyze in depth the provisioning process of IWSN standards and discuss security problems.

## 3. Analysis of Provisioning and Security Consideration

In this section, we will: (i) analyze provisioning procedures of the ISA 100.11a standard and ISA 100.11a-certified devices, Zigbee and WirelessHART standards, and discuss provisioning security-related research; (ii) compare and analyze provisioning procedures to derive security issues and security requirements to cope with them; and (iii) consider provisioning to enhance security.

### 3.1. Provisioning Methods

The provisioning process is the step in which a new device acquires the information needed to enter an IWSN. The new device to be provisioned is called the device being provisioned (DBP) and the IWSN that the DBP is to join is called the target network. When there are many IWSNs, the device in the unprovisioned state cannot join the target network because there is no information to identify which IWSN is the target network. In addition, there is no way to send and receive confidential information such as the master key, so it is not possible to perform security-guaranteed joins. Accordingly, the DBP must obtain network-related information and trust-related information through the provisioning process. The network-related information includes the IPv6 address of the target network. The trust-related information includes the symmetric key that will be used to ensure confidentiality during the network join process. Based on these basic concepts, each IWSN standard defines its own different provisioning process and provisioning information.

#### 3.1.1. Provisioning Process of ISA 100.11a

IEC 62734, “Industrial networks—Wireless communication network and communication—ISA 100.11a”, is the latest version of the ISA 100.11a standard. We will analyze the provisioning procedure within this standard since this document is the most current.

IEC 62734 presents various techniques that can be used to acquire provisioning information and describes the provisioning process that applies these techniques to various network configurations. It proposes factory pre-provisioned, out-of-band (OOB), and over-the-air (OTA) provisioning techniques for acquiring trust-related information and proposes OOB and OTA provisioning techniques for acquiring network-related information. The network configurations are divided by the presence or absence of a provisioning device (PD) and CA system. The PD is a network device that is separated from the target network and is responsible for the provisioning process between the target network and the DBP. This allows the target network to communicate directly only with a trusted device, the PD, and avoid direct communication with the DBP that is not yet trusted. The provisioning information is divided into network-related information and trust-related information, and IEC 62734 classifies these two types of information as follows:

- Network-related information: network ID and bitmask, IPv6 address of the system manager, data link configuration (superframe information, channel information, etc.).
- Trust-related information: symmetric key for join, extended unique identifier (EUI)-64 address for security manager, network join method.

Network-related information only interferes with DBP’s network join process if forgery is performed. DBP will not be able to join to the target network, but in order to maintain the effect of an attack, the DBP must be under a continuous forgery attack. Compared to the required ability and cost of the attacker, the effect of this kind of attack is not severe. So, it is believed that the security importance of network-related information is relatively lower than trust-related information. ISA 100.11a standard uses a well-known K_global key for transmitting the network-related information. Therefore, in this paper, rather than dealing with all provisioning processes available depending on their situations, we focus on trust-related information in order to analyze the security of the provisioning process. Therefore, we analyze the factory pre-provisioned, OOB, and OTA techniques that can be used to obtain trust-related information and the procedures for obtaining trust-related information using these techniques.

Factory pre-provisioned means literally the case that the information necessary for the join process is input at the manufacturer and distributed to the user. After a device is manufactured, the basic device with default information is in an unprovisioned state. The default information includes unique address, EUI-64 address, and global/open key. The state that the asymmetric key pair with the certificate or the symmetric key is entered in the unprovisioned state is the factory pre-provisioned state. The security key entered in a device must also be entered in the whitelist of the security manager, which is a device responsible for the security of a target network. The standard does not define the method for inputting this information to the security manager, but suggests methods such as email, CD, and keyboard. The certificate should be made out based on the CA information of a target network.

The OOB technique uses another secure communication line. The standard does not define the detailed OOB procedure, but suggests methods such as infrared communication, wired connectors, and keyboards on devices. Therefore, though the terms are different, factory pre-provisioned can also be seen as a kind of OOB.

The OTA technique does not use other communication lines but the existing network to perform communication over the air. There are asymmetric and symmetric key-based OTAs. The asymmetric key-based OTA is used when the DBP has an asymmetric key and certificate but the target network does not support an asymmetric key-based join process. In this situation, the DBP needs a new symmetric key to perform the symmetric key-based join process. The new symmetric key is encrypted using the DBP’s public key and transmitted to the DBP. On the other hand, the symmetric key-based OTA is used when the CA is built and the target network also supports the asymmetric key-based join process but the DBP does not support the asymmetric key. In this case, the DBP should perform symmetric key-based provisioning and requires a symmetric key. If the DBP does not have a previously entered symmetric key using OOB or factory pre-provisioning, OTA communication is performed using the default symmetric key, K_open. When obtaining provisioning information using K_open, trust-related information can be leaked through eavesdropping because K_open is a well-known key.

**Figure 1:**
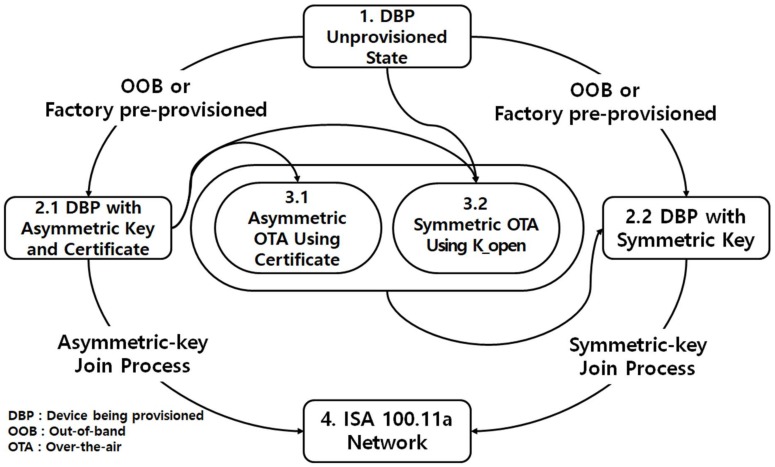
Acquisition of trust-related information as per ISA 100.11a-IEC62734.

Trust-related information can be obtained by using these three techniques, as shown in Figure 1. The provisioning process can be divided into five procedures.

- 1→2.1→4: Case that DBP has been issued a public key and certificate with OOB or is factory pre-provisioned, and target network supports asymmetric key-based join process.
- 1→2.2→4: Case that DBP is issued a symmetric key with OOB or is factory pre-provisioned, and target network supports symmetric key-based join process.
- 1→2.1→3.1→2.2→4: Case that DBP is issued a public key and certificate with OOB or is factory pre-provisioned but new symmetric key must be issued because target network does not support asymmetric key join process, and PD can perform asymmetric key-based operation.
- 1→2.1→3.2→2.2→4: Case that DBP has been issued a public key and certificate as OOB or is factory pre-provisioned but a new symmetric key must be issued because the target network does not support the asymmetric key join process, and the PD cannot perform the asymmetric key-based operation.
- 1→3.2→2.2→4: Case that DBP does not have pre-issued trust-related information.

The fourth and fifth procedures acquire the symmetric key using the well-known K_open key, which would allow an attacker to decrypt the encrypted symmetric key via eavesdropping. Therefore, the fourth and fifth procedures should be used only in an environment secure from eavesdropping.

#### 3.1.2. Provisioning Process of ISA 100.11a-Certified Devices

There are currently 52 products from 17 companies [24] that are certified by the ISA 100 Wireless Compliance Institute. We analyze the provisioning process that is in use by Company A. For the ISA 100.11a product from A, provisioning is performed through a USB device using wireless communication. Provisioning information is uploaded to the USB device through software provided by company A. Based on the input information, the provisioning process is performed with DBP using OTA, but all provisioning information including the trust-related information, is transmitted in plaintext. Therefore, even though Company A’s product has a certification from the ISA 100 wireless compliance institute, Company A’s provisioning process is vulnerable. Company A’s products do not use the provisioning procedures based on the security suggested in the standard. It is believed that this is because the standard only suggests various provisioning methods without enforcing a requirement.

#### 3.1.3. Provisioning Process for Zigbee and WirelessHART

Zigbee and WirelessHART are other prominent IWSN standards. Zigbee is the first standard that implements the upper layers based on IEEE 802.15.4, but it has disadvantages in that it does not support channel hopping and does not provide the necessary scalability. Nevertheless, because various IWSN studies using Zigbee have been carried out, we analyze the latest standard, Zigbee Pro [2]. WirelessHART was the first IWSN international specification approved by IEC and has been widely used together with existing HART equipment since the 1980s; we analyze the latest standard document [8].

In the case of Zigbee, the provisioning information is the only master key, and acquisition of network-related information is not required because it performs the join to the nearby network device. There are two ways to obtain the master key: the factory pre-provisioned method and the method of receiving the unencrypted keys distributed through a trust center, which is a device responsible for key distribution. As a result, security relies on factory pre-provisioning.

In the case of WirelessHART, the provisioning information is a join key and network ID of the target network. WirelessHART relies on a handheld device carried by a person in a plant. Therefore, the WirelessHART device needs a “maintenance port” to communicate with the handheld device and performs the provisioning by performing wired/wireless communication with the handheld device through the maintenance port. A direct wired provisioning process using a handheld device can be seen as a kind of OOB. In the case of wireless communication, there is a restriction on the physical distance between the handheld device and DBP because the standard sets a limitation of one-hop communication between the handheld device and the DBP for security reasons. There is also a restriction that session keys must be shared in advance for secure communication between the handheld device and DBP but the method for this is not described in the standard. Therefore, it can be summarized that WirelessHART devices depend on OOB communication between the handheld device and the maintenance port of the DBP.

#### 3.1.4. Provisioning Process of Park and Lee and Chen et al.

We analyzed the related work of provisioning process methods by Park and Lee and Chen et al. Since there is no provisioning function in the IEEE 802.15.4 layer, each IEEE 802.15.4-based standard presents a different provisioning process and data structure. The method of Park and Lee proposed an elliptic curve cryptography (ECC)-based provisioning procedure available in the IEEE 802.15.4-based Low-Power Wireless Personal Area Networks (LoWPAN) specification and defined the IEEE 802.15.4 frame for this. Therefore, it can be used in all IEEE 802.15.4-based standards, including Zigbee, WirelessHART, and ISA 100.11a, regardless of differences in the IEEE 802.15.4 higher layer, and the proposed procedure is as follows:

(1) DBP and PD generate an ECC-based asymmetric key.
(2) The PD stores the public key of the DBP and media access control (MAC) address.
(3) DBP performs ECC-based asymmetric key join process, and PD performs authentication of the DBP through information stored in step 2.

The method of Park and Lee does not differ significantly from the OOB method because the device responsible for provisioning, such as the PD, must have the ECC public key and MAC address in advance. ECC also can be used in ISA 100.11a, which performs access control via an EUI-64 address in the join process. Therefore, the method of Park and Lee differs from the provisioning method of ISA 100.11a in that it authenticates with the public key of ECC instead of a certificate of CA.

The method of Chen et al. is also an ECC-based provisioning procedure similar to that of Park and Lee. However, unlike the method of Park and Lee, CA-based authentication was used for authentication and its validity was verified by applying it to WirelessHART. Their method used HART communication foundation (HCF) instead of building an additional system responsible for the CA role. However, as it uses the CA system, there exists a restriction that keying material related to the CA must be distributed in advance. The method of Chen et al. differs from other provisioning methods in that the DBP has the key creation capability itself. Therefore, while other provisioning methods require additional security measures for the situation when a key exchange is required due to leakage of a security key, the method of Chen et al. can remotely respond effectively by performing the provisioning process again.

### 3.2. Security of Provisioning Process

As ISA 100.11a is a specification designed for IWSN, it suggests various provisioning techniques and procedures. In addition to ISA 100.11a, we analyzed various provisioning processes including ISA 100.11a-certified devices, other prominent IWSN standards such as Zigbee and WirelessHART, and the methods of Park and Lee and Chen et al.

IWSN specifications provide various secure provisioning processes, but there are preconditions required for each procedure. If the preconditions are not satisfied, an unsecure provisioning process should be used, and the techniques vary from plaintext transmission to encryption communication using well-known keys to ensure minimum integrity.

The security of IWSN provisioning processes, including ISA 100.11a, relies on OOB or factory pre-provisioned information. According to a technical report on factory pre-provisioning [25] published by Zillner, the master key of Zigbee Light Link (ZLL), one of the specifications using Zigbee Pro, was leaked. Therefore, it cannot be said that factory pre-provisioned products are free from security incidents. Also, as described in the provisioning procedure of ISA 100.11a, if factory pre-provisioned information cannot be used, security cannot be provided because a new key must be issued. The OOB technique using handheld devices in WirelessHART does not have a security issue, but it is difficult to use it in a large scale IWSN because its physical limitations make it inefficient. Therefore, a provisioning procedure having low-cost and simplified operational procedures is needed.

There are other two provisioning process problems. First, it is impossible to perform a key update because the generation of a new secret key is impossible when core security key information is leaked from a security-managing device of a network, such as the security manager of ISA 100.11a and trust center of Zigbee. The method of Chen et al. may be a measure to overcome this problem because in their method, the DBP can generate a key by itself without OOB or being factory pre-provisioned. The second problem is with multiple networks because the provisioning process cannot perform provisioning by selecting a proper network since there is no network-related information. The following are requirements of the provisioning process, which are derived from analysis of the security issues in the provisioning process:

Provisioning process issues:

- Even though a procedure cannot obtain trust-related information through OOB and factory pre-provisioning, it must be a secure procedure.
- Unlike provisioning procedures that use WirelessHART handheld devices, it must be a low-cost, simplified operation procedure that can be used in a large-scale IWSN.
- The end device must be able to generate a new key value in preparation for the leakage of trust-related information, which is the root of the security key generation.
- Even in multi-network situations, provisioning of the target network should be able to be performed.

There exist several ways to enhance the security of provisioning. As an example, the following security-enhancing provisioning techniques can be used to satisfy the security requirements to solve the security problem of the provisioning process:

Security enhancing provisioning techniques:

- ECDH-based key establishment: Algorithms for key generation and exchange are required for the DBP to generate its own security key. ECC was chosen because it has a small sized-key and is more efficient than other asymmetric key-based techniques [26] due to the nature of sensor nodes with memory constraints. In addition, the ECDH-based key exchange scheme was selected by selecting the Diffie–Hellman (DH) scheme because the asymmetric key-based method in which public keys should be distributed in advance could not be used in order to avoid the technique that requires pre-shared information.
- EUI-64 address whitelist-based authentication: Authentication is performed through the EUI-64 address of a device in order to solve authentication, which is a security vulnerability of the DH-based key exchange technique. There are two requirements for this. First, the PD or the device responsible for provisioning must have EUI-64-based whitelist information of communication-allowing devices. Second, the IWSN requires additional security techniques to complement EUI-64-based authentication, such as physical access control on the IWSN.
- Solicitation and advertisement: Generally, when a new device is to communicate with the IWSN, it waits to receive advertisement messages. The advertisement messages include time information for synchronization and channel information when frequency hopping is performed. The new device receives these advertisement messages, synchronizes the network, and performs communication in the proper way. However, because there is no network-related information in the DBP, if there are multiple IWSNs, the DBP cannot determine which advertisement message is the target network’s advertisement message. Therefore, in this paper, we propose a solicitation and advertisement method in which the DBP sends a provisioning request message first and then the advertisement device (PD) checks the pre-stored EUI-64 address whitelist; then, only the advertisement device of the appropriate network for the DBP transmits the advertisement message to the DBP.

This paper focuses on analyzing the provisioning process, which is very important for security in deployment of an IWSN and verifying the security issues through experiment. Therefore, Section 4 verifies security issues for the provisioning process in standards and certified devices, and also security enhancing provisioning techniques that can be considered a method for provisioning security.

## 4. Case Studies and Discussion

Among the IWSN standards, WirelessHART relies on handheld equipment and Zigbee relies on factory pre-provisioned information for provisioning process security. Handheld equipment has limitations in large-scale IWSN management and the factory pre-provisioned method cannot guarantee security because the security level relies on the factory, which is outside of the IWSN. Zillner’s technical report also shows factory pre-provisioned devices have security issues. Moreover, a detailed provisioning process with handheld equipment or factory provisioning is not described in the standards. Therefore, we focus on methods that do not depend on handheld equipment or factory pre-provisioned information. As a result, the provisioning processes of Zigbee and WirelessHART are excluded, and, among the provisioning processes of ISA 100.11a, the OTA provisioning process using K_open is selected as an experimental case.

Thus, in this section, we verify the security issue of the provisioning process through the case study of the ISA 100.11a standard’s OTA provisioning process using K_open and the provisioning process of an ISA 100.11a-certified device. We discuss the importance of security enhancement for the provisioning process and its solution, including a case study of ISA 100.11a security-enhancing provisioning, which can be one of the methods used to secure the provisioning process.

### 4.1. Case A under ISA 100.11a Standard Specification

#### 4.1.1. Test Environment

The test environment used to verify the ISA 100.11a standard’s OTA provisioning process using K-open is as follows:

- DBP: NXP MC1322x sensor node
- PD: NXP MC1322x network node
- Compile: IAR embedded workbench 3.0
- Firmware loader: Freescale MC1322x firmware loader
- Source code: NIVIS ISA 100.11a open source code
- Packet capture: Beamlogic site analyzer and Wireshark
- Debugging: Teraterm

We used IEEE 802.15.4-based products and sniffer equipment. For the DBP and PD, NXP’s MC1322x sensor node and MC1322x network node were used, respectively. MC1322x devices have a specification of a 32-bit ARM7TDMI-S core, 128KB flash memory, and 80KB ROM. The IAR embedded workbench was used to develop and generate the binary file which was uploaded to the MC1322x devices through the Freescale MC1322x firmware loader. The code used for development is the ISA 100.11a open source code developed by NIVIS until 2013 [27], in which the provisioning process was not implemented. We implemented the OTA provisioning process using K_open by using necessary related code. Since ISA 100.11a performs communication in 16 frequency bands through channel hopping, packets cannot be captured with a general IEEE 802.15.4 sniffer because it captures only one channel. Therefore, we tested the developed codes by capturing 16 IEEE 802.15.4 frequency bands at once using Beamlogic’s 802.15.4 site analyzer. Debugging was performed by outputting the message via Teraterm using the USB port of the MC1322x devices.

**Figure 2:**
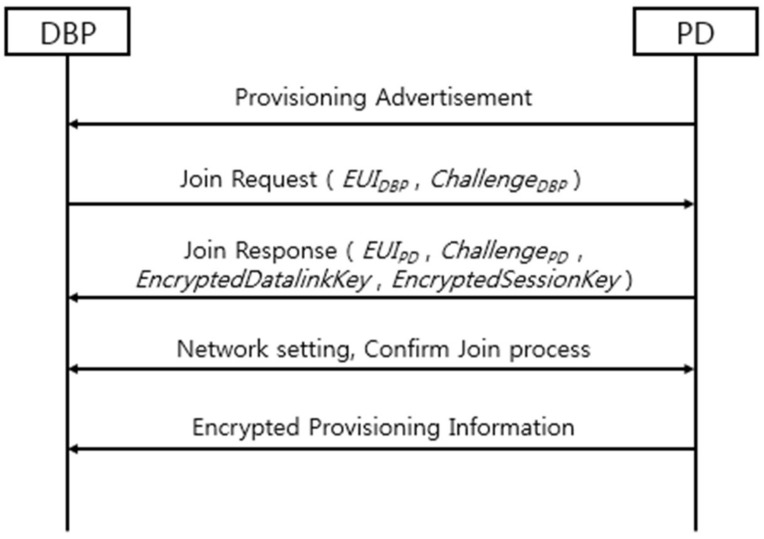
Sequence diagram for the ISA 100.11a standard’s over-the-air (OTA) provisioning process using K_open.

Figure 2 is a sequence diagram of ISA 100.11a standard’s OTA provisioning process using K_open. The DBP performs ISA 100.11a standard’s join process with the PD using K_open. The DBP starts the join process by transmitting the join request message with DBP’s EUI-64 address and 16-byte random challenge value. Then, the PD creates the master key using the received DBP’s EUI-64 address and challenge value, and also its own EUI-64 address and challenge value. Equation (1) shows how PD creates the master key. HMAC_MMO(K)_(M) means the message M’s hash-based message authentication code (HMAC) using Matyas–Meyer–Oseas (MMO) with K as an input key. The || symbol means concatenation, and EUI_A and Challenge_A mean EUI-64 address of device A and 16-byte challenge value of device A, respectively. Now the PD has a master key, a PD encrypt session key, and a datalink key, and can transmit to the DBP. Equations (2) and (3) show the encrypted session key and encrypted data link key. AES-CCM*_(K,N)_(M) means encrypted message of M using advanced encryption standard with counter with cipher-block chaining star (AES-CCM*) mode with K as an input key and N as nonce. For stable communication, there are network setting steps and confirming join process steps through hashed join messages. Finally, the DBP and PD can communicate with each other, so the PD transmits encrypted provisioning information using AES-CCM* with a session key as a secret key and current time value as a nonce. After DBP receives this information, it leaves PD’s network:

*MasterKey = HMAC_MMO(K_open)_(EUI_DBP||EUI_PD||Challenge_DBP||Challenge_PD)* (1)

EncryptedSessionKey = AES-CCM*_(Masterkey, Challenge_PD)_(SessionKey) (2)

EncryptedDatalinkKey = AES-CCM*_(Masterkey, Challenge_PD)_(DatalinkKey) (3)

EncryptedDatalinkKey = AES-CCM*_(SessionKey, Nonce)_(Provisioning Information) (4)

#### 4.1.2. Experiment and Security Analysis

**Figure 3:**
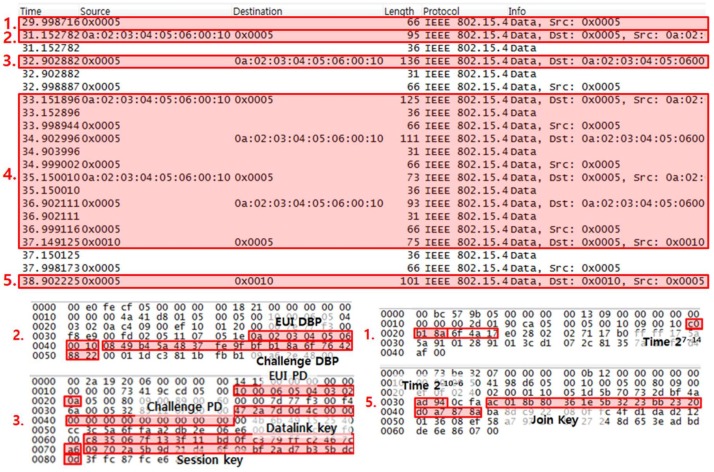
Captured packet of OTA provisioning process using K_open.

Figure 3 is a captured packet of the OTA provisioning process using K_open. Figure 3’s first red-boxed packet is the provisioning advertisement message, the second red-boxed packet is the join request message, the third red-boxed packet is the join response message, the fourth red-boxed packets are network setting and confirming join process messages and the fifth red-boxed packet is the provisioning information message. Under the assumption that the attacker has the ability to capture these packets through eavesdropping, we will show how an attacker can exploit transmitted join key (0x100F 0E0D 0C0B 0A09 0807 0605 0403 0201) in captured packets.

(1) Exploiting master key: As shown in Figure 3’s second and third red-boxed packet, DBP’s EUI-64 address and challenge and PD’s EUI-64 address and challenge. Using Equation (1), a master key can be obtained.
(2) Exploiting session key: Using obtained master key and the most significant 13 bytes of PD’s challenge, attacker can decrypt encrypted session key. The encrypted session key is shown in Figure 3’s third red-boxed packet. The process of encryption and decryption of AES-CCM* is the same because the counter is the same, 1. Therefore, using Equation (2), attacker can exploit the session key.
(3) Exploiting join key: When an encrypted session is initiated, the nonce consists of the EUI-64 address and time value, not the challenge value. The nonce required time of the transport layer service data unit (TPDU) is generated at a granularity of 2^−10–6^ s. The TPDU contains time at a granularity of 2^−10–6^ s at transport header and the rest of time at a granularity of 2^7–21^ s should be acquired through the time value included in the advertisement message within the last 64 s. The attacker can use the first packet of Figure 3 to get the time at a granularity of 2^7–21^ s. Using Equation (4), an attacker can exploit the join key. Table 1 summarizes values of exploited keys and parameters for exploiting key.

Therefore, through the OTA provisioning process experiment using K_open, we verified an attacker can exploit the join key using the well-known K_open key. Then, an attacker could exploit the master key and other keys of the DBP through eavesdropping of the join process using the exploited join key. Therefore, the DBP loses the security of all communication and attacker can hijack the session of the DBP. As the experiment shows, when using an unsecured provisioning process, security cannot be guaranteed with any encryption technique and security technique. In addition, this experiment was conducted with only one PD, but in the case of multiple IWSN environments, there may be more than two PDs in the vicinity of the DBP. In this case, the DBP cannot determine which PD is the PD of the target network through the received provisioning advertisement message. Therefore, the provisioning process cannot be performed in this situation.

### 4.2. Case B under ISA 100.11a-Certified Devices

#### 4.2.1. Test environment

**Figure 4:**
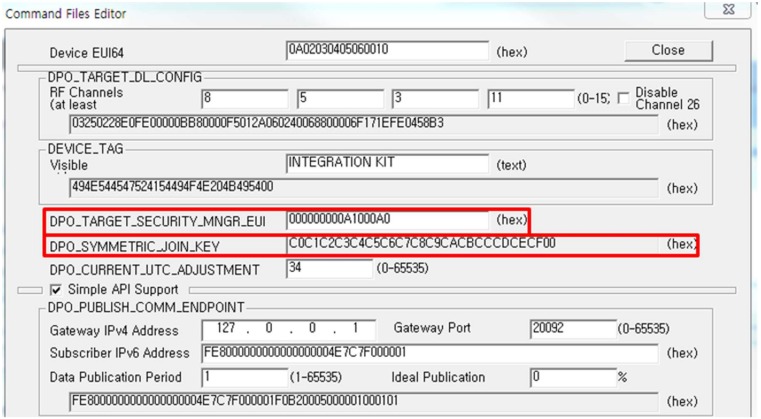
Configuration tool for the USB wireless provisioning device. The two red-boxed contents show the EUI-64 address of the security manager and the join key.

The ISA 100.11a products consist of a USB wireless provisioning device, two sensor nodes, and a gateway. The provisioning process is performed on the sensor node using the USB wireless provisioning device; the settings for the USB wireless provisioning device use the program of company A shown in Figure 4. Like case A, we used the multi-channel sniffer for capturing provisioning process packets.

#### 4.2.2. Provisioning Process of ISA 100.11a-Certified Devices

Before the provisioning process, we configured the provisioning information using the configuration tool for the USB wireless provisioning device. After that, the provisioning process can be performed. The USB wireless provisioning device sends the advertisement message periodically and when the DBP receives the advertisement message, it sends the provisioning request message to the USB wireless device. Then, the DBP receives the provisioning information in plaintext from the USB wireless provisioning device. The provisioning information is divided and sent separately, and DBP sends an acknowledgement (ACK) message when it receives each provisioning information message.

#### 4.2.3. Experiment and Security Analysis

**Figure 5:**
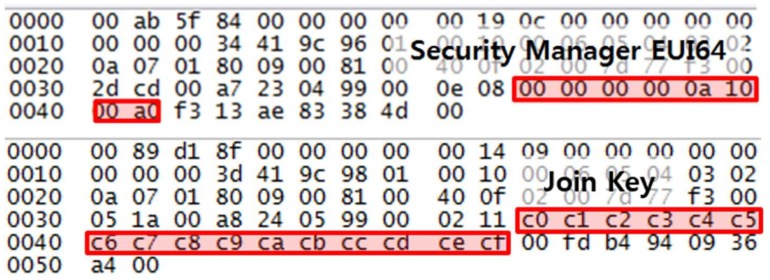
Captured provisioning packet of EUI-64 address of the security manager and the join key. The EUI-64 address of the security manager and the join key are the same as the contents in the Figure 4 red box.

All provisioning information is transmitted in plaintext, including the EUI-64 address of the security manager and the join key. Figure 4 shows the configuration tool of the USB wireless provisioning device; the EUI-64 address of the security manager and the join key are red-boxed. Figure 5 shows the captured provisioning packet of the EUI-64 address of the security manager and the join key which matches the plaintext configuration information in Figure 4. In the join process with a target network, unlike the join process of OTA provisioning, the EUI-64 address of the security manager is used instead of the EUI-64 address of the PD in Equation (3) to create the master key. Except for the EUI-64 address of the security manager and the join key, the other values of Equation (3) can be easily obtained by eavesdropping on the join request/response message. Therefore, the EUI-64 address of the security manager and the join key are classified as trust-related information in the ISA 100.11a standard, and confidentiality of this information is necessary. However, as shown in Figure 4 and Figure 5, the EUI-64 address of the security manager and the join key are transmitted in plaintext, so an attacker can obtain the master key using eavesdropping and Equation (1), and every security measure for the IWSN could become meaningless. In addition, the USB wireless provisioning device should be operated near the DBP to limit the communication distance to one hop, like the wireless handheld device of WirelessHART. Therefore, the provisioning process of an ISA 100.11a-certified device is a method which is difficult to use in a large-scale network.

### 4.3. Case C under Security Enhanced Provisioning

In this subsection, we describe the security enhancing provisioning techniques described in Section 3 to address the security issues of the provisioning process by implementing them in ISA 100.11a, and the implementation issues we experienced.

#### 4.3.1. Test Environment

The test environment for case C is similar to that of case A. However, due to test board hardware capacity limitations, only the ISA 100.11a functions required for case C verification are used. The IEEE 802.15.4 Mac Demo Applications source code, the basic code of ARM7 MAC Codebase 2.2.0 provided by Freescale Beekit, is used for the base code and Openssl-1.0.2l was used as the library of ECDH.

#### 4.3.2. Provisioning Process Using Security Enhancing Techniques on ISA 100.11a

We verified the security enhancing provisioning techniques by implementing them on ISA 100.11a; the implementation details are described in this section.

**Figure 6:**
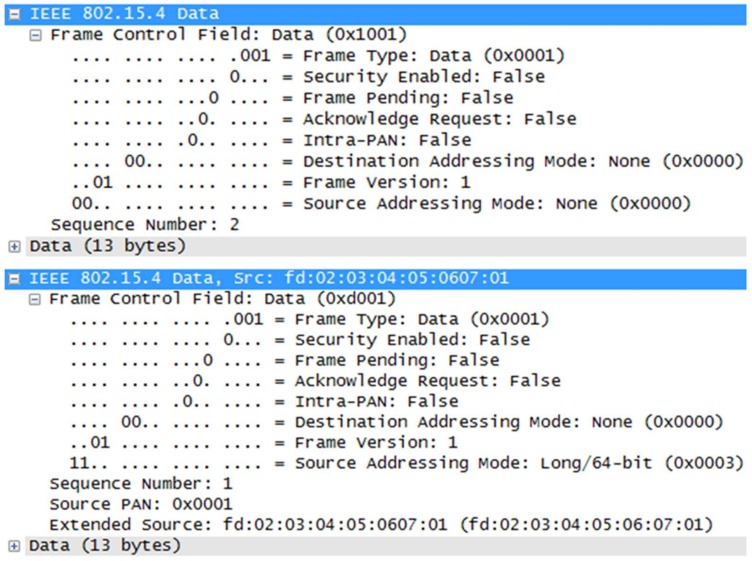
Solicitation message of ISA 100.11a standard (**top**) and case C (**bottom**). Solicitation message of ISA 100.11a standard does not include any address. However, case C needs the source address of the solicitation device for access control; thus, this message has compatibility issues with the ISA 100.11a standard.

- Solicitation-based provisioning request: The DBP without any provisioning information cannot perform normal communication with other devices because the DBP has no network information and time synchronization is not performed. In this situation, one option for the DBP is a solicitation message that the DBP can utilize to transmit the time value and the network value as 0. By transmitting a solicitation message, only the DBP-related PD sends an advertisement message to the DBP and the provisioning process can be initiated. However, there are two problems with this use of the solicitation message in case C. First, the solicitation message in the ISA 100.11a standard does not support addressing mode. In the case of the ISA 100.11a standard, the message structure without source and destination addresses is specified in the standard. However, in case C, the source addressing mode 3 is used because the EUI-64 address transmission is required for authentication. Figure 6 shows ISA 100.11a standard’s solicitation message (left) and case C’s solicitation message (right), which is not compatible with the ISA 100.11a standard. Second, there are additional requirements to use the solicitation method. Prior to the solicitation method, the ISA 100.11a standard [9] says, “Due to regulatory and safety requirements, some applications cannot tolerate data-link entities (DLEs) that transmit data-link protocol data units (DPDUs) while they are idle or in transit”. However, the solicitation message has the problem that an unauthorized device that has not yet been provisioned uses the DLE of the existing equipment. It means malicious node can sabotage existing equipment by sending many solicitation messages. Therefore, in the ISA 100.11a standard, it is described that even if the solicitation method is used, a method of enabling/disabling the solicitation method according to radio silence, radio sleep, and superframe idle state is required. As a result, based on inquiries to the ISA 100.11a product vendor, the solicitation method is not currently used in the ISA 100.11a IWSN. However, case C used a solicitation method that sends a solicitation message, including the EUI-64 address of the DBP to address the provisioning process’s issues, but a technique for managing the solicitation method should be further studied.
- EUI-64 address whitelist-based provisioning response: The PD of ISA 100.11a receives provisioning related information from the target network and the PD stores this information in the device provisioning service object (DPSO) to perform the provisioning process with the DBP. The first attribute of DPSO is the EUI-64 address array called White_List: the array of EUI-64 addresses of DPBs that are allowed to perform provisioning. Therefore, in case C, we used White_List to authenticate the DBP transmitted solicitation request. The DBP shall perform network synchronization and receive the parameters for generating the ECDH key through the advertisement message. There is an advertisement message in the ISA 100.11a standard that can be used for network synchronization and there is also a flag to distinguish it from the advertisement of the join process and the provisioning process. Therefore, the existing advertisement message is used in case C. However, since the existing advertisement method is transmitted within the other message, the source address/destination address cannot be used for the advertisement message. Therefore, if two or more devices simultaneously perform provisioning within the same vicinity, multiple provisioning requests/responses are generated, and thus proper provisioning cannot be performed. To solve this problem, case C uses a method of sending only the advertisement message of the provisioning response. However, it must be considered that the timing of the advertisement response should not be a timeslot allocated for other purposes to send only the provisioning response message and the previously mentioned solicitation requirement.
- ECDH-based encryption: The DBP and PD exchange the ECDH public key to generate the ECDH secret key. Using the openssl-1.0.2l library, we created the Curve P-384-based key, which is the cipher suite of the National Institute of Standards and Technology (NIST)—Commercial National Security Algorithm (CNSA). By performing the ECDH public key exchange, a shared secret key is generated by Equation (5) using the properties of elliptic curves:

PrivateKey_A_ * PublicKey_B_ = PrivateKey_B_ * PublicKey_A_ (5)

The generated ECDH symmetric key is 384 bits. Since the join key of ISA 100.11a is 128 bit, 1st–128th bit of ECDH symmetric key can be used as is or hashed for the join key. On the other hand, in the join process, end-to-end security can be guaranteed through the join key. However, according to the network configuration, the hop-by-hop security must use a global data link key because there is no secret data link key. In order to avoid the use of a global data link key, future research on ECDH-based provisioning processes according to network configuration can be carried out.

#### 4.3.3. Security Analysis

The provisioning process of case C has the key creation capability of the DBP using ECDH. Also, the DBP sends the solicitation and only the PD of the appropriate target network transmits a provisioning advertisement. Thus, the provisioning process can be performed on the appropriate target network among multiple IWSNs even if there is no pre-provisioned network information. Therefore, even if the provisioning information is not acquired through OOB or factory pre-provisioning, provisioning can be performed through ECDH and solicitation. In addition, provisioning is performed through OTA communication rather than use of a handheld device so it can be efficiently used in a large-scale network. Also, even if a security key is leaked, it is possible to rebuild security by generating a new key from the DBP side. However, it would be necessary to use a supplementary security measure, such as a CA system or physical access control, for authentication because case C uses only the EUI-64 address for authentication. If the authentication is guaranteed, the security issue of the provisioning process can be enhanced by the ECDH and solicitation method. Thus, this kind of research on provisioning processes, suitable for various IWSN environments, is needed.

### 4.4. Discussion

In case A, we verified the security issue of the provisioning process without using OOB and factory pre-provisioning by experimenting using ISA 100.11a standard’s OTA provisioning process using K_open. In case B, we emphasized the problem of the provisioning process and the need for research on its security by experimenting with the provisioning process of ISA 100.11a-certified devices. In case C, we verified the provisioning process using the ECDH and solicitation method by applying it to ISA 100.11a as one of the solutions to cope with the problems shown in the provisioning process through cases A and B.

Each secure provisioning process requires different preconditions and has different advantages. Therefore, it is necessary to analyze the security issues according to the IWSN environment that performs the provisioning process and to research countermeasures to cope with those security issues. In addition, solicitation or ECDH techniques can be a countermeasure against security issues, as confirmed in case C in the application of ISA 100.11a, but studies on revision and application of standards should be accompanied by verification of the countermeasures.

## 5. Conclusions

Because the provisioning process is the starting point of IWSN security and security leakage of the provisioning process makes all subsequent security steps meaningless, the security of the provisioning process is important, even in order to activate IIoT in the industrial domain. To this end, various provisioning processes have been proposed in many IWSN standards, including ISA 100.11a and existing studies, but the security of the provisioning process is still not sufficiently verified or guaranteed. This paper analyzed IWSN standards, including Zigbee, WirelessHART, and ISA 100.11a, and described their lack of security. Among the IWSN standards, the provisioning process of ISA 100.11a is well structured compared to other standards, so we focused on ISA 100.11a to examine security issues in the provisioning process and verified them through experimentation. As a result, first, there is no security provisioning procedure for low-cost and simplified operation procedures in which the DBP in unprovisioned state does not depend on OOB and factory pre-provisioning. Second, a new secret key cannot be generated, therefore there is no security key usable if the main key value is leaked. Lastly, because the DBP in the unprovisioned state does not have network-related information, in the case of multi-networks the DBP cannot perform the provisioning process by identifying the target network by itself. These security issues were verified through experiments on the OTA provisioning process using K_open in ISA 100.11a, which does not depend on OOB and factory pre-provisioning and an ISA 100.11a-certified device. Along with this, the security enhancing provisioning technique using the ECDH and solicitation scheme was proposed as one to be considered for the security of the provisioning process. The measures to cope with the security issue of the provisioning process and to enhance security were discussed. Future research needs to be conducted to apply the issues analyzed and verified through our experiments to an actual environment for the provisioning process security of IWSN for IIoT.

## Figures and Tables

**Table 1:** Value of exploited keys and parameters.

Type	Hex Value
K_open	0x004F00500045004E0000000000000000
EUI DBP	0x0A02030405060010
EUI PD	0x100006050403020A
Challenge DBP	0x0849B45A4837FE9FBFB18A6F76428822
Challenge PD	0x472A7D0D4C0000000000000000000000
Equation (1) = Master Key	0xB18A6F4A17E02802027117B0FFFF175A
Encrypted Session Key	0x09702A5B9D21D46F09BF2AD7B35BDC0D
Equation (2) = Session Key	0x0102030405060708090A0B0C0D0E0F10
EUI PD + Time (Nonce)	0x100006050403020AC72AAD94FF
Encrypted Join Key	0xAC018B80361E5B3223BB2320D0A7878A
Equation (4) = Join Key	0x100F0E0D0C0B0A090807060504030201
